# Feasibility and Safety of Percutaneous Axillary Artery Access in a Prospective Series of 100 Complex Aortic and Aortoiliac Interventions

**DOI:** 10.3390/jcm12051959

**Published:** 2023-03-01

**Authors:** Tim Wittig, Arsen Sabanov, Andrej Schmidt, Dierk Scheinert, Sabine Steiner, Daniela Branzan

**Affiliations:** 1 Department of Angiology, University Hospital Leipzig, 04103 Leipzig, Germany; 2 Helmholtz Institute for Metabolic, Obesity and Vascular Research (HI-MAG) of the Helmholtz Center Munich at the University of Leipzig and University Hospital Leipzig, 04103 Leipzig, Germany; 3 Department of Vascular Surgery, University Hospital Leipzig, 04103 Leipzig, Germany

**Keywords:** aortic aneurysm, endovascular intervention, complex endovascular aneurysm repair, upper extremity access, percutaneous closure device

## Abstract

We aimed to review the feasibility and safe use of the percutaneous axillary artery (AxA, 100 patients) approach for endovascular repair (ER) of thoraco-abdominal aortic aneurysms (TAAA, 90 patients) using fenestrated, branched, and chimney stent grafts and other complex endovascular procedures (10 patients) necessitating AxA access. Percutaneous puncture of the AxA in its third segment was performed using sheaths sized between 6 to 14F. For closing puncture sites greater than 8F, two Perclose ProGlide percutaneous vascular closure devices (PVCDs) (Abbott Vascular, Santa Clara, CA, USA) were deployed in the pre-close technique. The median maximum diameter of the AxA in the third segment was 7.27 mm (range 4.50–10.80). Device success, defined as successful hemostasis by PVCD, was reported in 92 patients (92.0%). As recently reported results in the first 40 patients suggested that adverse events, including vessel stenosis or occlusion, occurred only in cases with a diameter of the AxA < 5 mm, in all subsequent 60 cases AxA access was restricted to a vessel diameter ≥ 5 mm. In this late group, no hemodynamic impairment of the AxA occurred except in six early cases below this diameter threshold, all of which could be repaired by endovascular measures. Overall mortality at 30 days was 8%. In conclusion, percutaneous approach of the AxA in its third segment is feasible and represents a safe alternative access to open access for complex endovascular aorto-iliac procedures. Complications are rare, especially if the maximum diameter of the access vessel (AxA) is ≥5 mm.

## 1. Introduction

Endovascular aortic repair has become a preferred strategy for the treatment of thoraco-abdominal aortic aneurysm (TAAA) because of the substantially lower peri-operative risk compared to open surgery [1]. However, in many cases, delivery of bridging and visceral stents necessitates an upper extremity access (UEA), which might also be required to stabilize the graft during the procedure via a through and through wire or in other aortoiliac interventions, such as delivery of iliac branch devices (IBD). UAE can be performed by a variety of techniques using open cut-down or percutaneous access, and four sites are usually suggested for access to the upper extremities: the distal brachial artery on the inside of the elbow, the brachial artery on the medial humerus, the proximal brachial artery just below the axillary grove, and the infraclavicular approach via the axillary fossa [2]. Compared to the brachial or radial approach, the axillary artery (AxA) can be used for large sheath sizes > 7F and successfully accommodation of sheath sizes up to 18F via the AxA have been reported in patients undergoing transcatheter aortic valve replacement [3].

So far, there are no clear guidelines for the best approach, but it depends on the clinical circumstances as well as operator’s experience and preferences. Previous series providing results for both open and percutaneous access to upper extremity arteries reported overall low complication rates, but only a small number of percutaneous approaches were considered [2,4,5,6]. In case of percutaneous UAE, several approaches to closure have also been proposed, including manual compression and the use of closure devices.

We recently reported a preliminary series comprising 40 patients undergoing percutaneous axillary artery (AxA) access in the third segment with subsequent closure using the the Perclose ProGlide percutaneous vascular closure device (PVCD) (Abbott Vascular, Santa Clara, CA, USA) [7]. Importantly, results suggested that adverse events, including vessel stenosis or occlusion, occurred only in cases with a diameter of the AxA < 5 mm, and thus in all subsequent 60 cases AxA access was restricted to patients with a vessel diameter ≥ 5 mm. Here we report now the results of the full series of 100 patients undergoing percutaneous AxA access.

## 2. Material and Methods

### 2.1. Study Design and Patient Selection

This single-center cohort study enrolled 100 consecutive patients between September 2013 and November 2020 who required upper extremity access for endovascular aortoiliac procedures, primarily for TAAA with fenestrated, branched, and chimney stent grafts. In all patients, a percutaneous axillary approach with a 6–14F sheath was established in the third segment of the vessel under ultrasound guidance. Based on an interim analysis of the first 40 patients, complications at the puncture site were only seen when the maximum diameter of the axillary artery was <5 mm [7]. In all subsequent patients, percutaneous axillary access was only performed if the maximum diameter was ≥5 mm based on CT scan measurements. Eight patients were excluded from the study due to AxA diameter < 5 mm. There were no other exclusion criteria for percutaneous AxA access. The patients excluded for percutaneous AxA access were alternatively treated using a bilateral percutaneous brachial access. The Institutional Review Board of University of Leipzig approved the analysis of this data set obtained from a prospectively maintained aortic database.

### 2.2. Interventional Details and Postoperative Management

All interventions were performed under general anesthesia in a hybrid operating room equipped with a Philips Allura Xper FD 20 X-ray imaging system (Philips Healthcare, Amsterdam, The Netherlands) except for one patient who was locally anesthetized. Details on the branched and fenestrated stent graft implantation technique has been described previously [6,7]. Duplex ultrasound and computed tomography angiography (CTA) scans were evaluated before the procedure to assess the patency of the AxA, its size, and the presence of disease. An AxA with an anterior wall free of calcification and with a minimum diameter of 4.5 mm for the first 40 patients and with a minimum diameter of 5.0 mm for the subsequent 60 patients was considered suitable for large bore vascular access. Under ultrasound guidance, the operator aimed to puncture the anterior wall of the AxA in its third segment. Puncture was performed between the lateral border of the pectoralis minor muscle and the inferior border of the teres major muscle to minimize the risk of pneumothorax and to have the option for manual compression of the vessel against the humeral head using an 18 G needle and standard J wire. Care was taken to avoid injury to the brachial plexus and axillary vein. Access via the left AxA was preferred to avoid manipulation of the aortic arch. After 5F sheath insertion, a baseline control angiogram was performed to confirm correct positioning before proceeding with subsequent interventional steps. Five thousand international units of heparin were administered intravenously and then adjusted in order to achieve an activated clotting time of 250 s. A 5 mm skin incision was made at the axillary puncture site and the subcutaneous tissue was circumferentially stretched to facilitate insertion of the PVCDs. Two Perclose ProGlide PVCDs (Abbott Vascular, Santa Clara, CA, USA) were positioned in a typical 90 degree-angle fashion followed by sheath exchanges to achieve the size required for the planned endovascular aortoiliac procedure.

In those 90 patients treated for TAAA, a femoro-axillary through and through wire was established using a 300 cm long Lunderquist Guidewire (Cook Medical Inc, Bloomington, IN, USA) for percutaneous insertion of the aortic component of the thoracoabdominal stent graft. In order to ensure early restoration of blood flow to the pelvis and to the lower limbs, the right femoral access was closed, and the left femoral access was downsized by exchanging the stent graft sheath with a 9F 11 cm long introducer. Hemostasis of femoral access was achieved by pulling the long suture of the PVCDs. As the next step, stenting of the of the visceral target vessels was performed via the axillary approach.

At the end of the procedure, the axillary sheath was removed before final left femoral access removal and the sutures of the PVCDs were tied down over a safety J wire in place, while applying manual compression on the AxA over the humeral head. Adequate hemostasis was monitored clinically and angiographically. No additional external hemostatic agents were used.

**Figure 1:**
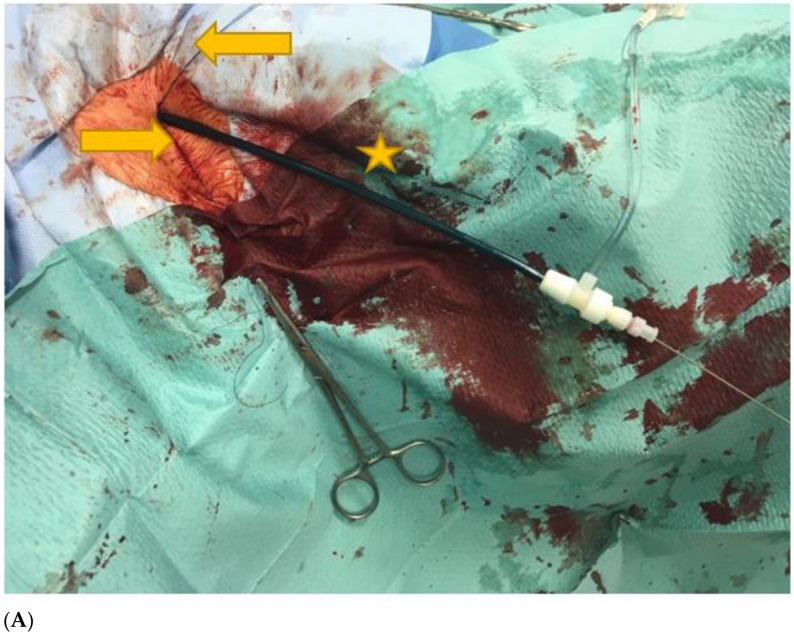

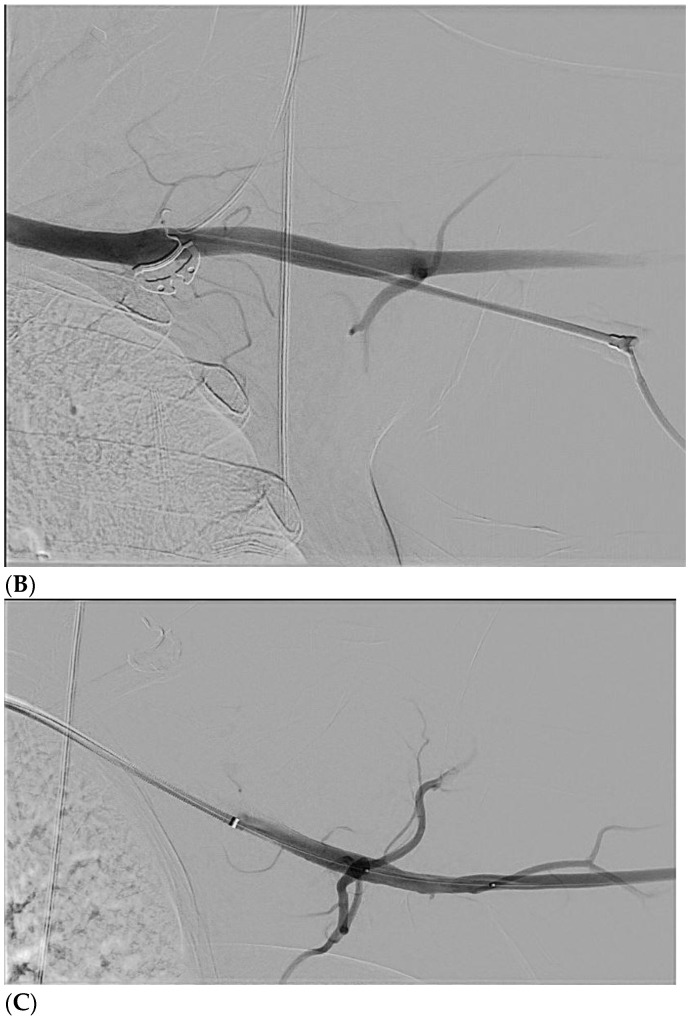
(**A**): Left axillary region. Puncture was made in the third segment of the AxA. The picture shows a 12F sheath (yellow star), which was introduced into the AxA. In addition, the two preloaded Perclose ProGlide PVCDs (Abbott Vascular, Santa Clara, CA, USA) (yellow arrows) can be seen. (**B**): Baseline angiography of the axillary artery (AxA) after ultrasound guided puncture in the third segment of the vessel. Consistent with the results of a previous CT scan, no relevant atherosclerotic changes were detected in this vessel segment, and the vessel diameter was >5 mm. The patient required pre-planned treatment for chronic thoraco-abdominal aortic aneurysm. (**C**): After successful fenestrated endovascular aortic repair (FEVAR) with fenestrations for the left and right renal artery, coeliac trunc, and superior mesenteric artery, closure of the axillary puncture site was performed using two Perclose ProGlide PVCDs (Abbott Vascular, Santa Clara, CA, USA). The control angiogram via the left femoral access showed no complications at the AxA puncture site.

A representative example of percutaneous AxA access and closure is presented in Figure 1A–C.

In patients with inadequate hemostasis, an additional PVCD was initially used, and if bleeding persisted, a covered self-expanding stent (Viabahn, W.L. Gore & Associates, Flagstaff, AZ, USA) was inserted into the AxA via the femoral approach after controlling proximal bleeding with semi-compliant balloon occlusion. In case of flow limiting dissection or occlusion, antegrade treatment of the injured vessel was performed by implantation of a self-expanding stent. The choice of the stent was at the discretion of the operator.

All patients under general anesthesia were transferred to an intensive care unit (ICU) for monitoring using a standardized post-operative management protocol and, if necessary, for further treatment. As part of clinical routine, all patients received a CT scan of the aorta as well as a duplex ultrasound of all puncture sites to identify access site complications before discharge.

### 2.3. Outcome Definitions

Device success was defined as successful puncture site closure using PVCD and no evidence of persistent bleeding or relevant hemodynamic impairment necessitating endovascular or open surgical repair. Procedural success was defined as establishing hemostasis and flow of the AxA using any endovascular method and freedom from major cerebrovascular and peripheral neurological complications by 48 h after the procedure. In addition, complications were recorded based on definitions from the Society for Vascular Surgery’s reporting standards for endovascular aortic aneurysm repair [8] and 30-day mortality was analyzed.

### 2.4. Statistical Analysis

All data were obtained from a prospectively maintained aortic database within our vascular center, in which imaging studies were evaluated using three-dimensional image analysis techniques (3Mensio Medical Imaging BV, Bilthoven, The Netherlands). Descriptive statistics were performed using SPSS version 20.0 (IBM, Armonk, NY, USA). Categorical variables are presented as number and percentages and continuous variables as mean ± SD or median values (range).

## 3. Results

### 3.1. Patient and Procedural Characteristics

Detailed patient demographics and characteristics of the AxA are presented in Table 1 and Table 2.

Included patients had a mean age of 73.8 ± 8.2 years and three quarters were male. Most procedures were performed for TAAA (90.0%). The mean aneurysm diameter of the TAAA cases was 65.7 mm (SD: 14.4 mm). Further characteristics of the treated TAAA are listed in Table 3. Endovascular TAAA treatment was pre-planned in 72 patients, while 18 patients presented as vascular emergencies: from those, 9 patients had an acute aortic rupture, 3 patients had a symptomatic penetrating aortic ulcer and 6 patients presented with acute, non-controllable pain.

Most patients were treated by branched endovascular aortic repair (BEVAR; 45%), followed by fenestrated endovascular aortic repair (FEVAR; 30%), chimney endovascular aortic repair (ChEVAR; 9%), and fenestrated-branched endovascular aortic repair (FBEVAR; 6%). The remaining 10% were mixed cases with different indications: a coil embolization of the superior gluteal artery because of endoleak Type II was performed twice via UEA. In two cases, an axillary approach was needed for bi-iliac extension after endovascular aortic repair (EVAR) and in four cases for delivery of iliac branch devices. One case each required UEA access for endovascular repair of renal artery bleeding and subclavian artery aneurysm (Table 4).

Based on CT scan measurements, the median diameter of the AxA in its third segment was 7.27 mm (range: 4.50–10.80). Most procedures were performed with a 12F introducer (60%), followed by 7F (18%), 8F (15%) and 9F (5%). Just in two single cases, a 6 and 14F introducer was used. None of the patients had significant calcification of the anterior wall of the vessel as examined in CTA. Except for one case, all patients were treated under general anesthesia. The mean operating time was 191.0 min (SD: 69.0) (Table 4).

### 3.2. Acute Procedural Outcomes

Device success was reached in 92 of the 100 patients (92.0%) using the two Perclose ProGlide PVCDs (Abbott Vascular, Santa Clara, CA, USA) inserted after puncture. Eight patients (8.0%) required additional procedures performed immediately during the index procedure via transfemoral access to either successfully close the puncture site (two patients) or restore adequate flow (six patients). Two bleedings occurred in the area of the puncture site despite use of closure devices, necessitating deployment of self-expanding covered stents. One of those patients exhibited a vessel diameter below 5 mm. In three patients, AxA occlusion occurred instantly after use of two PVCDs and was resolved in two cases by transfemoral implantation of 6 mm diameter self-expanding uncovered nitinol stents and by a 6 mm self-expanding covered stent in another patient. Furthermore, three patients experienced a high-grade stenosis at the percutaneous approach of the AxA, which was successfully repaired by implantation of self-expanding uncovered stents with a diameter of 6 mm. A total of six patients (6%) suffered stenosis or occlusion of the AxA at the puncture site. In all six cases with the above complications, the maximum diameter of the AxA was <5 mm. No cases required conversion to open surgical repair of the AxA.

Procedural success was achieved in 100% of the patients as there were no subsequent major vascular complications at the UEA access site requiring late reintervention or open surgery. No patient developed acute arm ischemia or deep arm vein thrombosis. Nine minor vessel complications (9.0%) were detected, including three pseudo-aneurysms and six hematomas, which all could be treated conservatively. No peripheral nerve injuries or brachial plexus damage were noted. Ischemia of the spinal cord occurred to varying degrees in 11 patients (11%) after the procedure. Seven patients also suffered a stroke (7%) and five of those events were considered peri-procedural based on neurologic assessment and CT scan. In three cases, symptoms completely resolved over the next few days and patients were asymptomatic at the time of discharge. Two patients with middle cerebral artery infarction had to be transferred to a neurological rehabilitation facility due to persisting hemiparesis and aphasia. The median ICU stay was 2 days and the median hospital stay 12 days (Table 5).

### 3.3. All-Cause Death through 30 Days

The thirty-day survival rate was 92%, but all deaths occurred during hospitalization. In four patients who died, the reason of death was aneurysm related. Three patients died within 24 h after endovascular treatment of ruptured TAAA and one because of a retrograde type A aortic dissection. Three patients developed multiorgan failure after the procedure, which could not be resolved despite intensive care measures. One patient became infected with SARS-CoV-2 virus after the endovascular procedure and died of COVID-related pneumonia with global respiratory insufficiency at day 30.

## 4. Discussion

As the complexity of endovascular aortic repairs has increased over time, the need for UEA has increased, particularly for bridging and visceral stent delivery, graft stabilization via a through and through wire, or complex aortoiliac procedures, such as IBD delivery. AxA access is often preferred over the brachial artery as the vessel can accommodate large sheath sizes up to 18F and is rarely atherosclerotic. To date, most surgeons prefer surgical exposure of the AxA, but the potential advantages of percutaneous access are shorter operation time and less vascular trauma with a lower risk of wound healing problems. However, only limited data are available on the safety of such an approach.

Our study aimed to characterize the feasibility and safety of percutaneous axillary artery access in its third segment in a series of 100 complex aortic and aortoiliac interventions with closure of the puncture site by percutaneous closure systems. While there are no specific vascular closure devices for the AxA, the use of two Proglide—deployed at the beginning of the procedure before sheath upsizing—has been suggested as a promising approach. The AxA can be divided into three segments based on anatomic structures [3]. Due to its deep submuscular location, the second segment is considered unsuitable for direct puncture. Its close location to the ribcage with the risk of pneumothorax in the context of puncture, as well as the lack of an adequate posterior bony structure for compression, are reasons against puncturing the first segment, and thus we decided to prefer the third segment of the AxA. Using this location, we report an overall 92% device success rate, which was even higher when we changed our practice after a preliminary analysis of the first 40 patients [7] excluding vessels below a 5 mm diameter threshold. Subsequently, no relevant stenosis or occlusion of the AxA was detected in the final control angiogram after puncture site closure. Bleeding events were rare (two cases), which also could be resolved using endovascular techniques. In no case was open surgical repair of the AxA required. Overall, our complication rate (e.g., occlusion/stenosis, bleeding, and hematoma) is comparable to similar studies in the field [3,8,9], but focusing on the excellent results of our late cohort, where we excluded vessel diameter < 5 mm, we actually report fewer complications. Importantly, we achieved these excellent results by using the third segment of the AxA, where the puncture risk can be considered lower due to the anatomical conditions, while most prior data were obtained after puncture of the first AxA segment. Bertoglio et al. investigated safety and effectiveness of UEA with percutaneous closure of the axillary artery (AxA) during endovascular treatment of TAA with fenestrated and branched endografts in 59 patients. They also used, for closing of the puncture site, the double ProGlide technique. In contrast to our study, the puncture of the AxA was carried out in the first segment using sheath sizes between 10 and 16F. The closure success rate was 90% with no open conversion required. A total of 5 out of 59 patients received a bare or covered stent implantation for either flow-limiting dissection or persistent bleeding. After a follow-up period of 6 months, there were no late complications, and all access vessels were patent [10]. Agrusa et al. investigated the safety and feasibility of a percutaneous AxA access also in the third segment in 46 patients with TAAA using two Perclose ProGlide devices (Abbott Vascular, Santa Clara, CA, USA) before inserting a large sheath. Technical success was achieved in 41 of 46 patients (89%) and 5 patients required endovascular covered stent implantation to control persistent access site bleeding. No surgical intervention was required in this cohort either [9].

Furthermore, Schaefer et al. reported percutaneous closure of AxA access in the first segment during transaxillary transcatheter aortic valve replacement in 100 patients and demonstrated the feasibility and safety of this approach as the rate of minor vascular complications was acceptable. Covered stent implantation was necessary in 11% of the cases [3].

Prior data from cardiac interventions also suggest that a percutaneous AxA access offers a similar safety compared to open cut-down. A systematic review studying patients undergoing transcatheter aortic valve replacement or mechanical circulatory support with large-bore axillary arterial access showed similar major vascular complications (2.8% vs. 2.3%) but less major bleeding (2.7% vs. 17.9%) using a percutaneous versus a surgical approach [11].

### Limitation

The limitations of the study include the sample size of this single center, non-controlled study, which did not allow for adjusted analysis. Further, effect sizes tend to be overestimated in non-controlled studies. Proving the feasibility and safety of the percutaneous axillary access would require a randomized controlled trial versus open surgery and other UEA sites. Long-term data would be needed to adequately assess the consequences after bailout stenting in case of occlusion/stenosis or bleeding complications with covered and uncovered stents.

## 5. Conclusions

Percutaneous puncture of the AxA in its third segment for insertion of large sheaths up to 14F in the percutaneous endovascular treatment of complex aortic and aortoiliac interventions seems to be safe, especially in arterial segments with a diameter > 5 mm. Complication management can also be performed with endovascular techniques.

## Figures and Tables

**Table 1:** Patient characteristics.

Variables	No.	%
Sex		
Male	75	75.0
Female	25	25.0
Age, years		
Mean ± SD	73.8 ± 8.2	
Median (range)	76.0 (54–87)	
History of hypertension	98	98.0
COPD	24	24.0
Active smoking	51	51.0
CHD	30	30.0
Diabetes mellitus	28	28.0
Chronic renal insufficiency *	68	68.0
Hyperlipidaemia	88	88.0
BMI (kg/m²)		
Mean ± SD	26.6 ± 4.6	
Median (range)	25.8 (20.0–42.4)	
PCI-pre-OP	10	10.0
CABG pre-OP	5	5.0
Creatinine pre-OP (µmol/L)		
Mean ± SD	125.3 ± 113.2	
Median (range)	95.5 (42.0–763.0)	
ASA Score		
ASA II	22	22.0
ASA III	77	77.0
ASA IV	1	1.0
Antiplatelets	81	81.0
Anticoagulant	21	21.0

Continuous data are presented as means ± SD; categorical data are given as counts (percentage). COPD = chronic obstructive pulmonary disease; CHD = coronary heart disease; BMI = body mass index; PCI = percutaneous coronary intervention; CABG = coronary artery bypass graft; ASA = American Society of Anesthesiologists; SD = standard deviation. * Defined as estimated glomerular filtration rate < 60 mL/min/1.73 m^2^.

**Table 2:** Characteristics of the AxA.

AxA Description Pre-OP		
Diameter AxA (mm)		
Mean ± SD	7.26 ± 1.29	
Median (range)	7.27 (4.50–10.80)	
Calcification > 50% circumference	0	0
Stenosis > 50%	0	0
Previous percutaneous access	0	0
Pacemaker on the punctured side	4	4.0
Dialysis AVF on the punctured side	5	5.0
Side of puncture AxA		
Left	93	93.0
Right	7	7.0

Continuous data are presented as means ± SD; categorical data are given as counts (percentage). AxA = axillary artery; AVF = arteriovenous fistula; SD = standard deviation.

**Table 3:** Characteristics of thoraco-abdominal aortic aneurysms.

Aneurysms Characteristics	No.	%
Acute	18	18.0
Rupture	9	9.0
Penetrating aortic ulcer	3	3.0
Pain	6	6.0
Chronic	72	72.0
Crawford Classification		
Type II	32	32.0
Type III	33	33.0
Type IV	25	25.0
Maximum aortic diameter, mm		
Mean ± SD	65.7 ± 14.4	
Median (range)	66.0 (25–102)	
Previous repair of the aorta	42	42.0
TEVAR	20	20.0
EVAR	22	22.0
TAAA		
Atherosclerotic	82	82.0
Dissection	8	8.0
Previous coil of segmental arteries	55	55.0

Continuous data are presented as means ± SD; categorical data are given as counts (percentage). TAAA = Thoraco-abdominal aortic aneurysms; SD = standard deviation; TEVAR = Thoracic endovascular aortic repair; EVAR = Endovascular aortic repair.

**Table 4:** Endovascular treatment characteristics.

Treatment Characteristics	No.	%
FEVAR (with no. of fenestrations)	30	30.0
2	5	5.0
4	25	25.0
BEVAR (with no. of branches)	45	45.0
2	3	3.0
3	7	7.0
4	34	34.0
5	1	1.0
FBEVAR (with no. of fenestrations)	6	6.0
4	5	5.0
5	1	1.0
ChEVAR (with no. of fenestrations)	9	9.0
3	3	3.0
4	5	5.0
5	1	1.0
Other	10	10.0
General anesthesia	99	99.0
Operative time (minutes)		
Mean ± SD	191.0 ± 69.0	
Median (range)	193 (53–480)	
Fluoroscopy time (minutes)		
Mean ± SD	50.3 ± 22.7	
Median (range)	49 (16–142)	
Radiation dose (Gycm²)		
Mean ± SD	1567.6 ± 2156.2	
Median (range)	429.7 (52.0–8635.6)	
ID of introducer (French)		
6F	1	1.0
7F	18	18.0
8F	15	15.0
9F	5	5.0
12F	60	60.0
14F	1	1.0
Median (range)	12 (6–14)	

Continuous data are presented as means ± SD; categorical data are given as counts (percentage). FEVAR = fenestrated endovascular aortic repair. BEVAR = branched endovascular aortic repair; FBEVAR = fenestrated-branched endovascular aortic repair; ChEVAR = chimney endovascular aortic repair; SD = standard deviation.

**Table 5:** In-hospital outcomes.

Variables	No.	%
Primary hemostasis AxA	92	92.0
Stenosis/occlusion AxA	6	6.0
Bleeding	2	2.0
Uncovered stent	5	5.0
Covered stent	3	3.0
Surgical repair	0	0
Hematoma	6	6.0
PSA	3	3.0
Arm ischemia	0	0
Peripheral nerve injury	0	0
DVT	0	0
Stroke	7	7.0
SCI	11	11.0
Death within 30 days	8	8.0
ICU stay (days)		
Median (range)	2 (0–34)	
Hospital stay (days)		
Median (range)	12 (1–95)	

Continuous data are presented as means ± SD; categorical data are given as counts (percentage). SD = standard deviation; AxA = axillary artery; PSA = pseudoaneurysm; DVT = deep vein thrombosis; SCI = spinal cord ischemia.

## Data Availability

The data presented in this study are available on request from the corresponding author. The data are not publicly available due to data privacy.

## Abbreviations

AxA	axillary artery
ER	endovascular repair
PVCD	percutaneous vascular closure devices
TAAA	thoraco-abdominal aortic aneurysms
UEA	upper extremity access
IBD	iliac branch devices
CTA	computer tomography angiography
BEVAR	branched endovascular aortic repair
FEVAR	fenestrated endovascular aortic repair
ChEVAR	chimney endovascular aortic repair
FBEVAR	fenestrated-branched endovascular aortic repair
EVAR	endovascular aortic repair
ICU	intensive care unit
